# Hedgehog Signaling in Tumor Cells Facilitates Osteoblast-Enhanced Osteolytic Metastases

**DOI:** 10.1371/journal.pone.0034374

**Published:** 2012-03-29

**Authors:** Shamik Das, J. Allan Tucker, Shikha Khullar, Rajeev S. Samant, Lalita A. Shevde

**Affiliations:** 1 Department of Oncologic Sciences, USA-Mitchell Cancer Institute, University of South Alabama, Mobile, Alabama, United States of America; 2 Department of Pathology, University of South Alabama, Mobile, Alabama, United States of America; 3 Department of Radiology, University of South Alabama, Mobile, Alabama, United States of America; Mayo Clinic College of Medicine, United States of America

## Abstract

The remodeling process in bone yields numerous cytokines and chemokines that mediate crosstalk between osteoblasts and osteoclasts and also serve to attract and support metastatic tumor cells. The metastatic tumor cells disturb the equilibrium in bone that manifests as skeletal complications. The Hedgehog (Hh) pathway plays an important role in skeletogenesis. We hypothesized that the Hh pathway mediates an interaction between tumor cells and osteoblasts and influences osteoblast differentiation in response to tumor cells. We have determined that breast tumor cells have an activated Hh pathway characterized by upregulation of the ligand, IHH and transcription factor GLI1. Breast cancer cells interact with osteoblasts and cause an enhanced differentiation of pre-osteoblasts to osteoblasts that express increased levels of the osteoclastogenesis factors, RANKL and PTHrP. There is sustained expression of osteoclast-promoting factors, RANKL and PTHrP, even after the osteoblast differentiation ceases and apoptosis sets in. Moreover, tumor cells that are deficient in Hh signaling are compromised in their ability to induce osteoblast differentiation and consequently are inefficient in causing osteolysis. The stimulation of osteoblast differentiation sets the stage for osteoclast differentiation and overall promotes osteolysis. Thus, in the process of developing newer therapeutic strategies against breast cancer metastasis to bone it would worthwhile to keep in mind the role of the Hh pathway in osteoblast differentiation in an otherwise predominant osteolytic phenomenon.

## Introduction

Bone homeostasis depends on the dynamic equilibrium between osteoblasts and osteoclasts and factors that mediate the crosstalk between them. Most malignant tumors, particularly breast and prostate cancers and even other tumor types such as thyroid, lung, and kidney preferentially metastasize to the skeleton [1]. Once in the bone, the tumor cells associate with the bone microenvironment and establish a functional entity that alters the balance coupling bone formation and bone resorption. These changes are brought about by cytokines and other growth factors produced by the metastatic tumor cells and can impact both, osteoclasts and osteoblasts. Some of the well-established factors expressed by tumor cells that impact bone resorption include TNF-α, -β, PTHrP, TGF-α, -β, CTGF, CXCR4, IL-11, MMP1 and OPN [2]. Signaling via the Hedgehog (Hh) pathway has been reported to upregulate the expression of PTHrP [3] by tumor cells leading to enhanced osteolysis [4].

In vertebrates the Hh pathway begins with the binding of Hh ligands (SHH, IHH or DHH) to the Patched receptors on the membrane. This relieves the inhibitory effect on Smoothened causing signal transduction into the cytoplasm that activates the GLI transcription factors to regulate transcription of target genes. This pathway stimulates osteoblast differentiation [5] and determination and differentiation of skeletal cells [6].

Our laboratory has recently shown that the Hh pathway plays a vital role in the crosstalk between breast cancer cells and osteoclasts [7]. In this study, we have dissected the role of the Hh pathway in the crosstalk between tumor cells and osteoblasts. We show that via Hh signaling the tumor cells facilitate osteoblast differentiation and deposition of mineralized matrix. These differentiated osteoblasts express RANKL, that together with OPN and PTHrP tilt the balance in favor of the osteoclasts. As such, our studies highlight the importance of the delicate balance between the activities of osteoblasts and osteoclasts and bring forth the importance of Hh signaling as an important attribute of the tumor cells' ability to cause osteolytic metastases.

## Materials and Methods

### Cell lines

Human fetal osteoblasts, hFOB 1.19 1.19 (ATCC, CRL-11372; obtained from ATCC, Manassas, VA) cells were cultured in Dulbecco's Modified Eagle's Medium (DMEM/F12; Invitrogen, Carlsbad, CA), supplemented with 2 mM L-glutamine, 1 mM sodium pyruvate, 0.02 mM nonessential amino acids, 5% FBS (Atlanta Biologicals, Norcross, GA), without antibiotics or antimycotics (DMEM/F12). MC3T3-E1 subclone 14 (ATCC, CRL-2594; obtained from ATCC) murine pre-osteoblast cells capable of differentiation and mineralization in culture (these lines exhibit high levels of osteoblast differentiation after growth in ascorbic acid and 3 to 4 mM inorganic phosphate) were maintained in alpha Minimum Essential Medium (αMEM) (Mediatech, Herndon, VA) and 10% FBS but devoid of ascorbic acid. RAW 264.7 (TIB 71; obtained from ATCC) cells, a murine preosteoclastic line capable of differentiation and mineralization in culture (in presence of RANKL and M-CSF) were grown in DMEM with L-glutamine (ATCC, 30-2002).

MDA-MB-231 human metastatic breast cancer cells, SUM1315 (derived from a metastasis in a patient with infiltrating ductal carcinoma), SUM159 cells (derived from a primary breast tumor with metaplastic carcinoma) and MDA-MB-435 (435) cells were cultured as described [7], [8]. The generation and culture conditions of 435 cells stably silenced for OPN (OPNi) or GLI1 (KO1 and KO4) is previously described [8], [9]. All cells were maintained in a humidified 5% CO_2_ environment at 37°C. All cultures were confirmed to be negative for *Mycoplasma* spp. infection using a PCR-based test (TaKaRa, Shiga, Japan). While the origin of the MDA-MB-435 cells is much debated, this cell line has been widely used to study the mechanisms and consequences of bone metastases and also to develop interventions in osteolytic metastasis [10], [11].

Harvesting conditioned medium: 3×10∧5 cells were seeded in 10 cm culture dishes and grown overnight in their respective complete media. The following day the cells were washed thrice in sterile PBS and growth media devoid of serum was added. This media was collected after 24 hours and used as conditioned media for the experiments.

#### Induction of osteoblastic and osteoclastic differentiation

In order to test the effect of conditioned medium from the tumor cells on osteoblast differentiation, a double-strength differentiation medium (DM) was formulated for MC3T3 E1 Sc-14 cells. It comprised αMEM, 20% FBS, 50 µg/ml ascorbic acid and 20 mM β-glycerophosphate. Conditioned media and the double-strength DM were mixed in a 1∶1 ratio. 1X DM was used as control. Similarly a double-strength differentiation medium was formulated for RAW 264.7 cell lines. It comprised 20% FBS, 50 ng/ml of RANKL (462-TR, R&D, Minneapolis, MN) and 20 ng/ml of M-CSF (M9160, Sigma, St. Louis, MO) added to the growth medium. Conditioned media from the tumor cells was mixed 1∶1 with the double-strength DM. 1X DM was used as control.

Osteoblast differentiation was assessed by alkaline phosphatase (ALP) activity assay in the perspective of total phosphatase which was done as previously described [11]. The functional assessment of osteoblast mineralization was done in triplicate quantified by staining with Alizarin Red S [3] and scoring the number of mineralized nodules in 12 independent fields per well.

### Apoptosis detection

MC3T3 cells were grown under differentiation conditions along with conditioned media from tumor cells for 21 days with media being changed every 3^rd^ day. At the end of 21 days apoptosis was assayed using the *In Situ* Cell Death Detection Kit (Roche, Indianapolis, IN) following the manufacturers' protocol for initial TUNEL staining. Cells were further stained with DAPI (Vectashield, H-1200, Vector Laboratories, Burlingame, CA) and phalloidin coupled with AlexaFluor 555 (Molecular Probes, Invitrogen) to visualize the nuclei and cytoskeleton respectively. The latter staining imparted context to the TUNEL staining. Cells were visualized under the Nikon TE2000 microscope and TUNEL positive cells were counted in 12 independent fields (in triplicate) and expressed as a percentage of total cells in each field of view.

### Western Blotting Analysis

Whole cell lysates were collected in NP-40 buffer (150 mM NaCl, 50 mM Tris, 1% NP-40). Isolation of cytosolic and nuclear fractions was done as previously reported [15]. Total protein (30 µg) was resolved by SDS-PAGE gel and transferred to PVDF membranes. Membranes were immunoblotted overnight at 4°C with antibodies to either OPN (Cat # 905-629; Assay Designs, Ann Arbor, MI) or GLI1 (sc-20687; Santa Cruz Biotech, Santa Cruz, CA). Equal loading was confirmed with anti-β-actin (A3854, Sigma) antibody. The purity of cytosolic and nuclear fractions was confirmed with either anti-β-tubulin (clone 9F3; cat#2128; Cell Signaling, Danvers, MA) or anti-HDAC1 (cat#2062; Cell Signaling) antibodies respectively. Secreted OPN was assessed by loading equal quantity of protein from the serum-free conditioned medium. Corresponding HRP conjugated secondary antibodies were used for detection; blots were developed with SuperSignal enhanced chemiluminescence substrate (Pierce, Rockford, IL) and imaged using a Fuji LAS3000 imager.

### Studies with Hh inhibitor, cyclopamine

Serum-free conditioned medium (SFM) harvested from ∼3.0×10^6^ cells after 24 hours was assayed for OPN by immunoblotting. To test the inhibitory effect of cyclopamine on the Hh pathway cells were cultured in DMEM supplemented with 0.5% FBS and treated for the indicated time intervals with DMSO (vehicle control) or cyclopamine (Sigma, St. Louis, MO).

### Luciferase Assay

Cells were transfected with the OPN promoter luciferase vector, pGL3-OPN either alone or in combination with pLNCX or pLNCX-GLI1 (kindly obtained from Dr. Jingwu Xie, University of Texas Medical Branch, Galveston, TX) as previously described [16]. Empty pGL3 vector was used as control. Hh ligands were added to the well 6 hours prior to harvesting the cells (∼33 hours of initiation of transfection) for assay. Readings were normalized to total protein content. Each parameter was studied in triplicate and the experiment repeated at least 3 times.

### Quantitative RT-PCR (qRT-PCR)

cDNA was generated using High Capacity Reverse Transcriptase Kit (Applied Biosystems, Foster City, CA). Real time PCR was performed using a BioRad iQ5 Real-Time Detection system (Bio-Rad, Hercules, CA). All reactions were done in triplicate. OPN transcript levels were normalized to GAPDH levels (dCT) which was used to calculate changes in OPN expression (2-ddCT). To analyze the effect of cyclopamine treatment on OPN expression untreated samples were set as calibrator (control) and compared to their respective treated samples. The primers used included Spp1 (OPN) (Mm 00436767_m1); Bglap (osteocalcin) (Mm 01741771_g1); IBSP (Mm 00492555_m1); PTHrP (Mm 00433057_m1); RANKL (Mm 00441906_m1); GAPDH (Mm 99999915_g1).

### Animal studies

One million MDA-MB-435 cells suspended in HBSS (Invitrogen) were injected into the left cardiac ventricle of 6-week old female athymic nude mice (Hsd: Athymic Nude-*Foxn1^nu^*; Harlan, Indianapolis, IN). The success of the intracardiac injection was assessed by the drawback of bright red arterial blood into the syringe immediately before and after injection, in contrast to venous injections evident by the darker colored blood [10]. Seven mice were scored for each group. Mice were euthanized 5 weeks later. Radiographic images were acquired using a small animal radiology equipment Acoma model PX 20N (72 KVP, 20 MA). The images were examined by ABR-Board certified radiologist (SK). Experiments were conducted under protocol approved by the Institutional Animal Care and Use Committee (IACUC) of the University of South Alabama, Mobile.

### Immunohistochemical analyses

We obtained breast tumor tissue microarrays from the NCI Cooperative Breast Cancer tissue Resource (CBCTR). The tissues were immunohistochemically stained for IHH and GLI1. Specifically, immunohistochemical staining was performed using the streptavidin biotin complex method with 3,3′-diaminobenzidine (DAB) chromogen using Dako LSAB+ System-HRP reagents (K4065) (which included a 3% hydrogen peroxide peroxidase block) in a Dako Autostainer Plus automated immunostainer (Glostrup, Denmark). Pretreatment for heat induced antigen retrieval was performed with a Thermo Fisher Scientific Lab Vision PT Module (Fremont, CA). Blocking for nonspecific binding was performed with an avidin/biotin blocking kit (SP-2001; Vector Laboratories, Burlingame, CA). The following antibodies were used: IHH (ab39634; Abcam, Cambridge, MA) and GLI1 (ab7523; Abcam). The intensity of cytoplasmic staining was quantitated with computer-assisted image analysis in a Dako ACIS III Image Analysis System (Glostrup, Denmark).

### Statistical Analysis

Statistical analyses were done using the t-test or ANOVA, per the mandates for each dataset, using GraphPad Prism 4 software. Statistical significance was determined if the analysis reached 95% confidence. In all figures the error bars represent standard error of the mean (S.E.M.). The exact p-values are listed in the corresponding figure legends.

## Results

### Expression of GLI1 and IHH is upregulated in breast cancer

**Figure 1:**
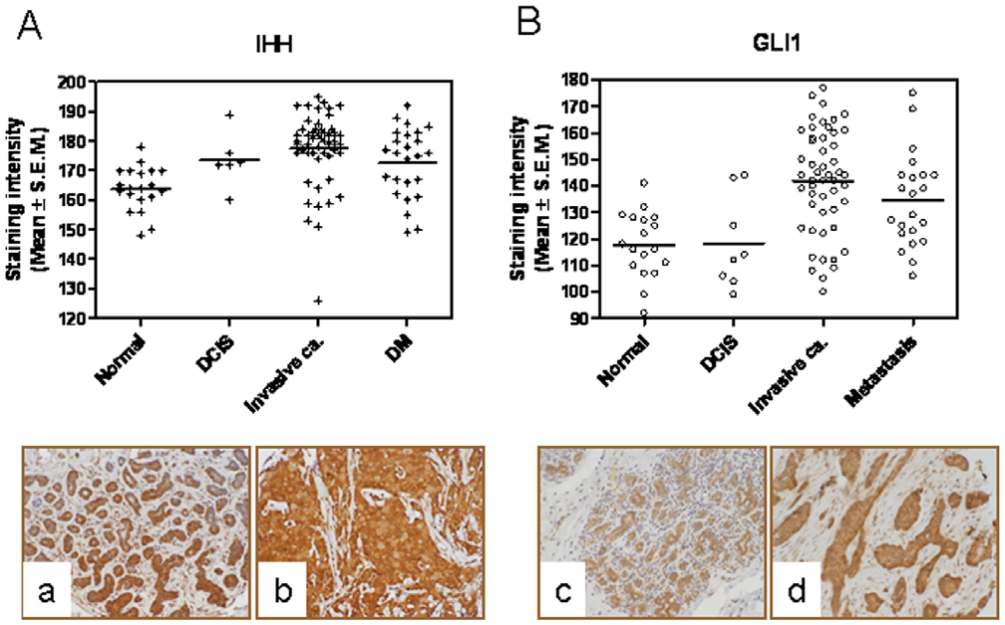
The Hh pathway is activated in breast tumors. Breast cancer tissues (n = 75) and normal breast tissues (n = 9) were immunohistochemically stained for (**A**) IHH and (**B**) GLI1 expression. The intensity of cytoplasmic staining was quantitated with computer-assisted image analysis in a Dako ACIS III Image Analysis System (Glostrup, Denmark). Staining intensities were recorded and represented as a scatter plot. The staining intensities indicating expression levels of IHH and GLI1 were significantly greater (p<0.0001) in the tissues derived from invasive cancer (representing Infiltrating Ductal Carcinoma Grades II–IV) and from metastatic breast cancer (DM) relative to normal tissues and tissues derived from Ductal Carcinoma In Situ (DCIS). Panels **a** and **b** represent normal breast tissue and invasive breast cancer stained for IHH. Panels **c** and **d** represent normal breast tissue and invasive breast cancer stained for GLI1.

Using immunohistochemical analyses, we assessed the expression of the Hh ligand, IHH and the transcription factor GLI1 in a tissue array comprising 75 breast cancer tissues and 9 tissues representing normal breast. While the staining intensity of IHH was comparable (p>0.05) in normal tissues and in tissues derived from Ductal Carcinoma In Situ (DCIS), the tissues derived from invasive cancer (representing Infiltrating Ductal Carcinoma Grades II–IV) and from metastatic breast cancer exhibited significantly (p<0.0001) increased staining intensity for IHH (**Figure 1A** **; images a and b**). Similarly, the staining intensity of GLI1 in tissues from invasive cancer and from metastatic cancer were significantly greater (p<0.0001) compared to normal tissues (**Figure 1B** **; images c and d**).

### Hh signaling stimulates osteoblast differentiation and mineralization activity

**Figure 2:**
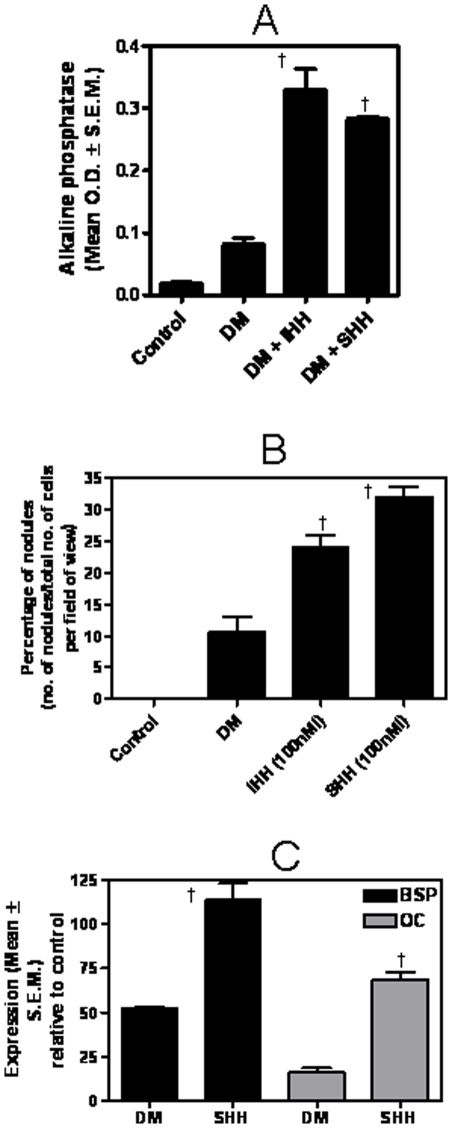
Activation of Hh signaling promotes differentiation and mineralization activity of osteoblasts. MC3T3 E1 Sc-14 cells were grown in differentiation medium (DM) supplemented with either with 100 nM recombinant SHH or IHH or without. Cells grown in normal growth medium were used as control. (**A**) Differentiation was assessed by alkaline phosphatase (ALP) assay. Under differentiation conditions that included recombinant IHH or SHH, the ALP activity was significantly greater († indicates p<0.0001) relative to DM alone. (**B**) Relative to control (growth medium), DM induced the formation of mineralized nodules. Relative to DM the media spiked with recombinant IHH and SHH supports the formation of significantly greater number of mineralized nodules († indicates p = 0.012 and 0.002, respectively). The number of nodules formed due to each treatment is represented as a percentage of the total number of cells present in each field of view. Control represents growth medium. The number of mineralized nodules in 12 independent fields per well were scored. (**C**) The expression of osteoblast differentiation marker genes, BSP († indicates p = 0.0027) and osteocalcin († indicates p = 0.0004) is significantly elevated in presence of SHH at the end of 14 days. Cells were harvested and RNA extracted which was used in real time quantitative RT-PCR assay. The fold change in expression is represented relative to control (growth medium).

In order to assess the effect of Hh signaling on the formation of osteoblasts, we used the monopotential cell line, MC3T3-E1, which is a clonal osteoblastic cell line isolated from calvariae of a late stage mouse embryo. These cells express various osteoblast functions including formation of mineralized bone nodules in long-term culture. The addition of Hh ligands, SHH and IHH to the DM of the MC3T3 cells stimulated differentiation as seen by the increase (p<0.0001) in the ALP activity (**Figure 2A**). The resultant osteoblasts exhibited intense staining by Alizarin Red S (**Figure S1A**) indicating the presence of mineralized nodules. Overall, we saw a significant increase (p<0.05) in the numbers of mineralized nodules formed in the presence of IHH and SHH (**Figure 2B**). This was accompanied by an increase (p<0.005) in the expression of markers of terminally differentiated osteoblasts [12], [13], bonesialoprotein (BSP) and osteocalcin (**Figure 2C**), indicating that stimulating Hh signaling promotes osteoblast differentiation and mineralization activity.

### Hh signaling upregulates OPN in osteoblasts

**Figure 3:**
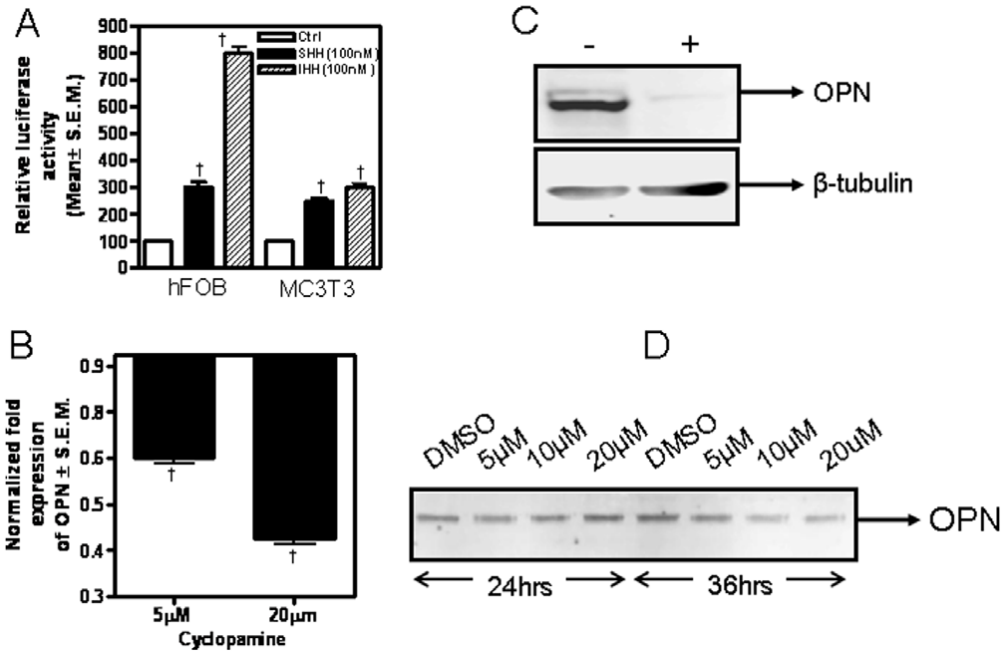
Hh signaling regulates OPN expression in osteoblastic cells. (**A**) Hh ligands significantly increase the activity of OPN promoter in the preosteoblastic cell lines hFOB 1.19 and MC3T3 E1 Sc-14 († indicates p<0.0001 for all indicated groups). Cells were transfected with OPN promoter, treated with Hh ligands at the indicated concentrations and assessed for luciferase activity. (**B**) The Hh pathway inhibitor, cyclopamine (20 µg/ml), decreased the expression of OPN transcript († indicates p<0.0001). hFOB 1.19 cells were treated with cyclopamine for 24 hours and assessed by quantitative real time RT-PCR for OPN expression. The expression of total OPN, from the cell lysate (**C**) as well as secreted OPN (**D**) is decreased in presence of cyclopamine. The decrease in the secreted OPN protein level is both, dose (5, 10 and 20 µM) and time dependent. hFOB 1.19 cells were treated with cyclopamine (20 µM for 24 hours; in panel C) and at theindicated concentrations and time intervals in panel D and assessed for expression of OPN by immunoblotting.

We have previously reported that Hh signaling induces the expression of OPN [8]. OPN promotes adhesion of osteoblasts allowing them to function in osteogenesis [14], [15], [16]. We treated two osteoblast-forming cells, hFOB 1.19 and MC3T3 with two Hh ligands, SHH and IHH and assessed the effect on OPN promoter activity. Both ligands caused an upregulation in OPN promoter activity (p<0.0001) (**Figure 3A**). Treatment with the Hh inhibitor, cyclopamine, keeps GLI1 sequestered in the cytosolic compartment (**Figures S1B & C**) simultaneous with a reduction in the levels of OPN transcript levels (p<0.0001) (**Figure 3B**), total OPN protein expression (**Figure 3C**) and secreted OPN (**Figure 3D**) in the pre-osteoblasts.

### Hh signaling in tumor cells stimulates differentiation of osteoblasts as an early event and enhances expression of RANKL and PTHrP

**Figure 4:**
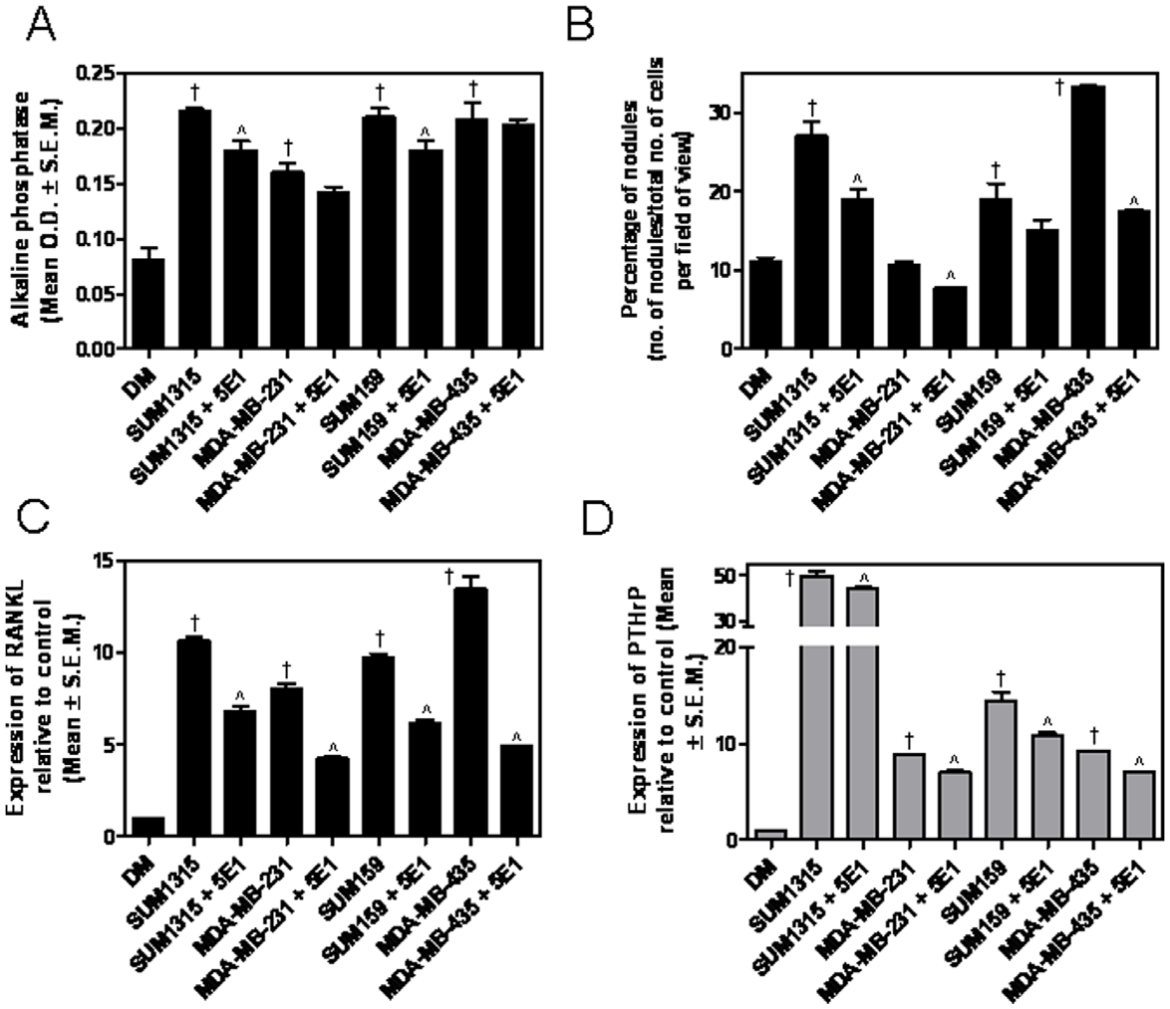
Hh ligands produced by tumor cells impacts the osteoblast differentiation and expression of RANKL and PTHrP. (**A**) Conditioned media from all four tumor cell lines caused a significant increase in ALP activity of MC3T3-derived osteoblasts: SUM1315 (†p = 0.0002); MDA-MB-231 (†p = 0.0035), SUM159 (†p = 0.0005), MDA-MB-435 (†p = 0.0009). Addition of the Hh neutralizing antibody, 5E1, caused a reduction in ALP activity. SUM1315+5E1 (∧p = 0.014); MDA-MB-231+5E1 (p = 0.12), SUM159+5E1 (∧p = 0.04), MDA-MB-435+5E1 (p = 0.6). (**B**) Tumor cell-conditioned media stimulated mineralization activity of the MC3T3-derived osteoblasts as evidenced by the numbers of mineralized nodules after Alizarin Red S staining. Nodules were counted in 12 independent fields and expressed as a percentage of the total number of cells in the field of view. SUM1315 (†p = 0.0013); MDA-MB-231 (p>0.05), SUM159 (†p = 0.018), MDA-MB-435 (†p<0.0001). Addition of the Hh neutralizing antibody caused a reduction in numbers of mineralized nodules. SUM1315+5E1 (∧p = 0.02); MDA-MB-231+5E1 (p = 0.0005), SUM159+5E1 (p = 0.17), MDA-MB-435+5E1 (p<0.0001). (**C**) The expression of RANKL by the differentiated MC3T3-derived osteoblasts was significantly increased in presence of tumor cell-conditioned medium. (†p = 0.0013, for all four tumor cell lines). Neutralization of the Hh ligand with the 5E1 antibody caused a reduction in the levels of RANKL expressed. SUM1315+5E1 (∧p = 0.0004); MDA-MB-231+5E1 (∧p = 0.0002), SUM159+5E1 (∧p = 0.0003), MDA-MB-435+5E1 (∧p = 0.0002). (**D**) The expression of PTHrP by the MC3T3-derived osteoblasts was notably greater in presence of conditioned medium from the tumor cells (p<0.0001 for all tumor cells). Neutralization of Hh ligand caused a significant decrease in the levels of PTHrP. SUM1315+5E1 (∧p = 0.04); MDA-MB-231+5E1 (∧p = 0.0001), SUM159+5E1 (∧p = 0.01), MDA-MB-435+5E1 (∧p<0.0001). The expression of RANKL and PTHrP were assessed by real time qRT-PCR after 14 days of differentiation. Data is expressed relative to control (growth medium).

Tumor cells express Hh ligands [7]. In order to determine the role of the Hh pathway in mediating the crosstalk between tumor cells and osteoblasts, we assessed the effect of conditioned medium from the tumor cells on MC3T3 osteoblast differentiation after 2 weeks using an ALP activity assay. Relative to DM alone, conditioned medium from the tumor cells caused a significant (p<0.001) increase in the ALP activity in 2 weeks. The 5E1 antibody blocks binding of all three mammalian Hh ligands to Ptc1 with low nanomolar affinity, thereby inhibiting Hh signaling [17]. Depleting the Hh ligands from the conditioned medium of the tumor cells using the neutralizing 5E1 antibody caused a decrease in the ALP activity of the differentiated osteoblasts. While the decrease was apparent, although not statistically significant with respect to the conditioned medium from MDA-MB-231 and MDA-MB-435 cells, the decrease was statistically significant (p<0.05) with respect to conditioned medium from SUM1315 and SUM159 cells (**Figure 4A**).

Simultaneous with the reduction in ALP activity, depletion of Hh ligands from the differentiation conditions caused a significant decrease (p<0.05) in the expression of (differentiated) osteoblastic proteins, BSP and osteocalcin (**Figure S1D**). Functionally, the ability of the osteoblasts to form mineralized nodules was significantly increased (p<0.0001) in response to conditioned medium from tumor cells relative to DM alone. Addition of the 5E1 antibody to the differentiation conditions resulted in a significant decrease (p<0.001) in the ability of the tumor cell-conditioned medium to elicit osteoblast mineralization activity (**Figure 4B**).

Differentiated osteoblasts express RANKL and PTHrP, both of which have been reported to play an important role in promoting osteoclast differentiation [18], [19]. Thus, we queried the expression of these two molecules under the conditions used for differentiation. In response to the conditioned medium from breast cancer cells, after 2 weeks of differentiation, the osteoblasts expressed significantly elevated (p<0.01) levels of RANKL and PTHrP (**Figure 4 C & D**). Depletion of Hh ligands from the conditioned medium of the tumor cells resulted in a significant decrease (p<0.001) in the levels of RANKL and PTHrP elicited by the conditioned medium. Thus, while Hh ligands from the tumor cell-conditioned medium contributed to osteoblast differentiation, their impact was more pronounced on the expression of RANKL and PTHrP by the differentiated osteoblasts.

### OPN expressed by the tumor cells influences osteoblast activity

**Figure 5:**
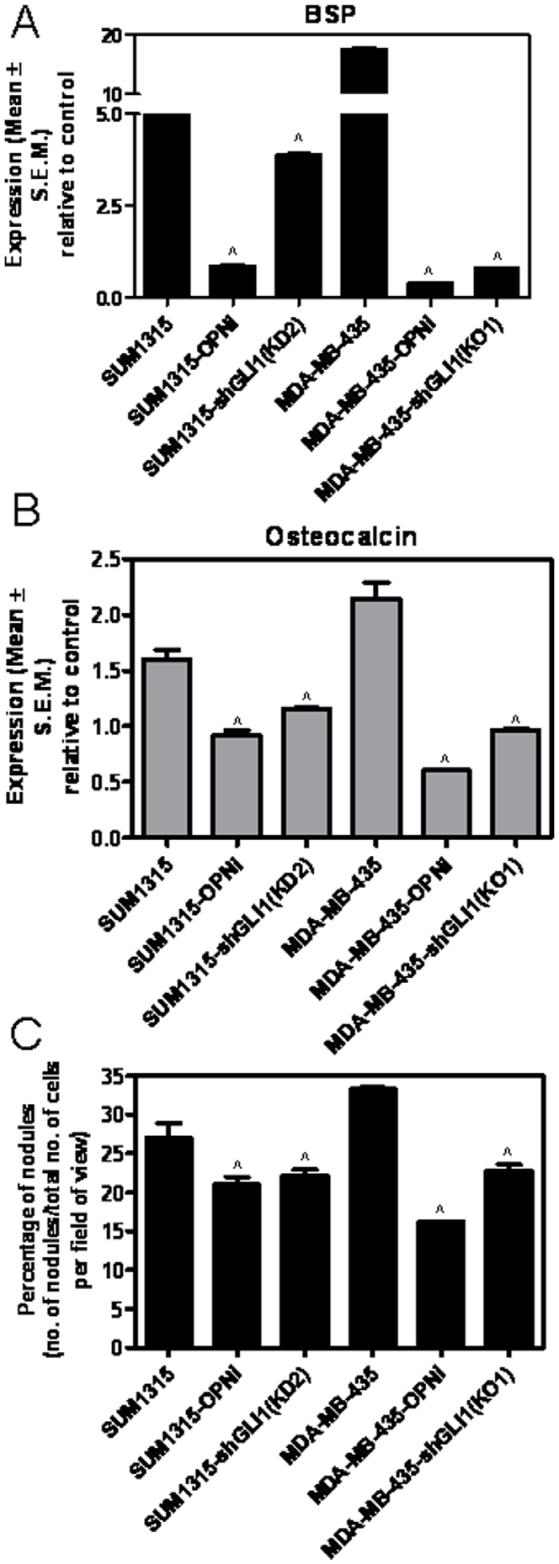
Tumor cells competent for Hh signaling and OPN expression are efficient at inducing osteoblast differentiation. Stable silencing of OPN (OPNi) or GLI1 (KD2 and KO1) from SUM1315 and MDA-MB-435 tumor cells significantly reduces the expression of (**A**) BSP [Relative to SUM1315, SUM1315-OPNi (∧p = 0.008) and KD2 (∧p = 0.0004) show lower BSP; Relative to MDA-MB-435, 435-OPNi and KO1 have decreased BSP (∧p<0.0001)], (**B**) osteocalcin [SUM1315-OPNi (∧p = 0.0013) and KD2 (∧p = 0.0004); 435-OPNi and KO1 (∧p<0.0001)], and (**C**) the mineralization capacity of the osteoblasts [SUM1315-OPNi (∧p = 0.04) and KD2 (∧p = 0.04); 435-OPNi (∧p<0.0001) and KO1 (∧p = 0.0003)]. The expression of BSP and osteocalcin were assessed by real time qRT-PCR and the nodules were quantified (in 12 independent fields in triplicate) after Alizarin Red S staining after 14 days of differentiation.

OPN, a secreted protein expressed by tumor cells, has been implicated as an important regulator of osteoblast differentiation [20]. Both, SUM1315 and MDA-MB-435 cells express OPN [7]. In order to assess the effect of tumor cell-derived OPN on the osteoblasts, we first abrogated OPN expression using shRNA [9] and harvested the cell-free conditioned medium from these cells. Osteoblast differentiation was studied in presence of this conditioned medium and we assessed the expression of BSP and osteocalcin as indicators of osteoblast differentiation and measured osteoblast differentiation activity by enumerating the mineralized nodules formed. The conditioned medium from the SUM1315-OPNi and 435-OPNi cells was less efficient (p<0.005) in inducing osteoblast differentiation and mineralization (**Figure 5 A–C**). Likewise, the expression of RANKL and PTHrP by the osteoblasts was significantly compromised (p<0.0001) under differentiation conditions with conditioned medium from tumor cell that were depleted of OPN expression (**Figure S2A**). As such, OPN expressed by the tumor cells plays a vital role in the crosstalk between tumor cells and osteoblasts.

### Hh signaling in tumor cells impacts their ability to induce osteoblast differentiation

We have recently published that Hh signaling in breast cancer cells also plays a vital role in communication between the breast cancer cells and osteoclasts [7]. In order to assess the role of Hh signaling in tumor cells on their ability to elicit osteoblast differentiation, we abrogated the expression of GLI1 from the tumor cells by shRNA [8]. Conditioned medium from the GLI1-silenced cells was inefficient (p<0.005) in inducing osteoblast differentiation as represented in the expression of BSP, osteocalcin (**Figure 5A & B**) and the osteoclast differentiation-promoting RANKL and PTHrP proteins (p<0.05) (**Figure S2A**). Further, the mineralization activity of the osteoblasts was also significantly impaired (p<0.05) when the differentiation was elicited for 2 weeks in presence of conditioned medium from cancer cells that were silenced for GLI1 (**Figure 5C**), suggesting that active Hh signaling in the tumor cells is vital to their ability to induce osteoblast differentiation.

### Extended differentiation in presence of tumor cell-conditioned media promotes osteoblast apoptosis

**Figure 6:**
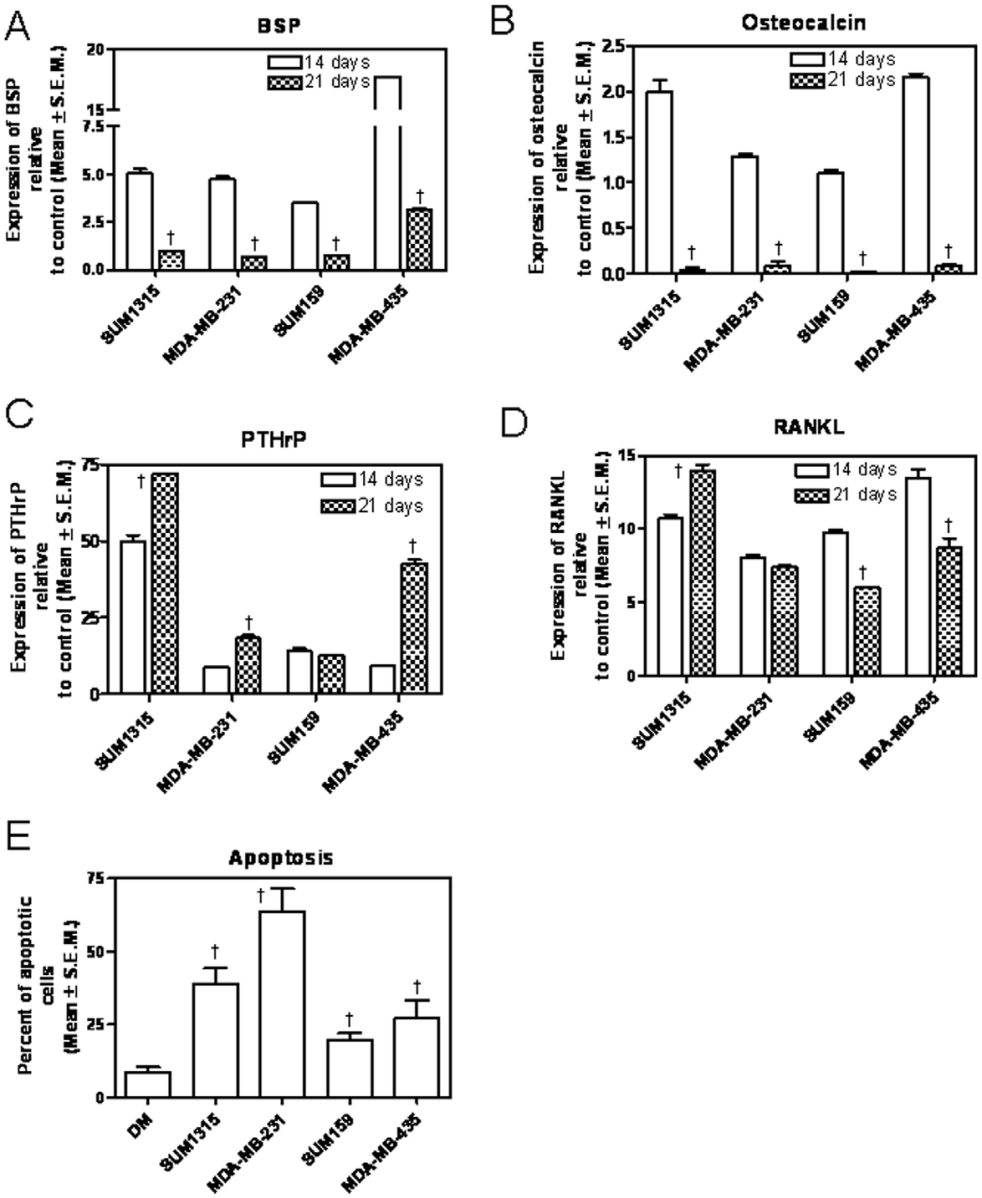
Extended differentiation in presence of tumor cell-conditioned media preserves RANKL and PTHrP expression but promotes osteoblast apoptosis. MC3T3 cells were grown in differentiation supplemented with conditioned media from cancer cells for either 14 days or 21days. At the end of each time point RNA was harvested from the differentiated osteoblastic cells and levels of BSP, osteocalcin, PTHrP and RANKL were assessed by qRT-PCR. (**A**) There is a significant decrease in the levels of BSP at 21 days relative to 14 days of differentiation for all four tumor cell lines assessed (†p<0.0001 for all tumor cells). (**B**)The levels of osteocalcin significantly decrease at 21 days relative to 14 days of differentiation (†p<0.0001 for all tumor cells). (**C**) The levels of PTHrP remained at elevated levels at 21 days post-initiation of differentiation (SUM1315: †p = 0.0005; MDA-MB-231: †p = 0.0006; SUM159: p>0.05; MDA-MB-435: †p<0.0001). (**D**) The levels of RANKL also remained elevated 21 days after differentiation. (SUM1315: †p = 0.0023; MDA-MB-231: p = 0.07; SUM159: †p = 0.0002; MDA-MB-435: †p = 0.004). The levels of RANKL and PTHrP were assessed by qRT-PCR. (**E**) Assessment of apoptosis was done at the end of 21 days post initiation of differentiation. Fluorescein conjugated TUNEL staining was performed to assay for apoptosis followed by nuclear staining with DAPI and cytoskeleton staining with phalloidin. Percentage of apoptotic cells was calculated as the number of cells with green fluorescence in the nucleus divided by the total number of cells (represented by the blue DAPI stain) in each field of view. Enhanced apoptosis of osteoblasts was noted in presence of conditioned media from all tumor cells (SUM1315 (†p = 0.005), MDA-MB-231 (†p = 0.002), SUM159 (†p = 0.01), MDA-MB-435 (†p = 0.04)).

Thus far, our data suggests that soluble factors that include OPN and the Hh ligands secreted by tumor cells enhance osteoblast differentiation and mineralization activity. This starkly contradicts the well-established fact that tumor cells causes osteoblasts to undergo apoptosis [11]. Notably, these reported studies conducted osteoblast differentiation for longer time periods i.e. 3 weeks or longer. Thus, in order to capture the full impact of the differentiation conditions on the osteoblasts, we conducted parallel experiments that were assessed 3 weeks post induction of differentiation. While differentiation and mineralization activity were already attained at 14 days, the levels of BSP and osteocalcin plummeted sharply (p<0.001) at 3 weeks relative to their expression at 2 weeks in differentiation conditions comprising conditioned media from tumor cells (**Figure 6A & B**). In contrast, the expression of PTHrP significantly increased (p<0.001) in presence of conditioned medium from 3 of the 4 tumor cell lines, whereas RANKL showed variation in the all the four cell systems investigated (**Figure 6C & D**). We also assessed the incidence of apoptosis following 21 days of differentiation in the presence of conditioned media from tumor cells. Relative to DM alone, the conditioned medium from all four tumor cells caused a significant increase (p<0.05) in the incidence of apoptosis (**Figure 6E** **& Figure S2B**), thus corroborating with the published reports. Thus, the data suggests that osteoblasts express osteoclastogenic factors, PTHrP and RANKL in response to OPN and Hh signaling triggered by tumor cells (**Figures 4** **,** **5** **& Figure S2A**).

### Hh signaling in tumor cells enhances the incidence and intensity of osteolytic metastases

**Figure 7:**
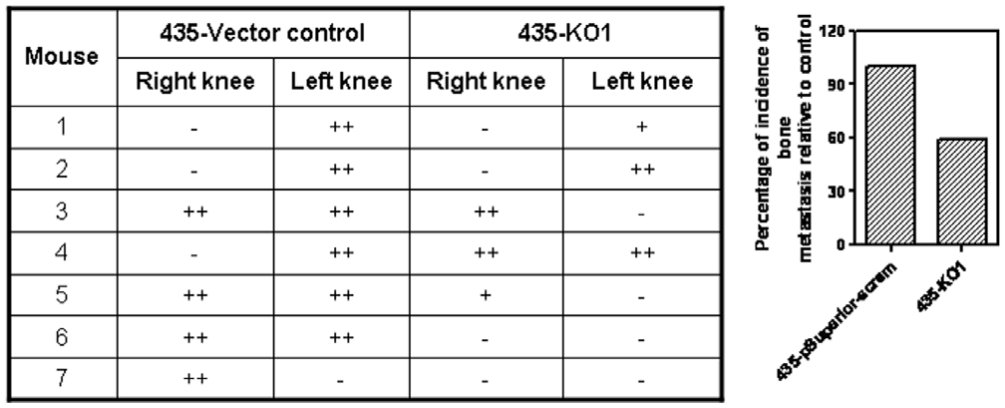
Active Hh signaling in the tumor cells is vital to their ability to cause osteolysis. MDA-MB-435 tumor cells were injected into the left cardiac ventricle of athymic mice; mice were euthanized 4–6 weeks later and radiographically imaged and assessed for osteolysis at the tibio-femoral junction. As represented in the Table, cells that were silenced for GLI1 expression showed an attenuated ability of osteolysis. The percent incidence of osteolysis is depicted in the adjacent graph.

Intuitively, our data suggests that tumor cells initiate osteoblast differentiation and the expression of osteoclastogenic factors as an early event, followed by elimination of osteoblasts later. Thus, the overall microenvironment appears to shift in favor of osteoclastogenesis. In order to investigate the significance of Hh signaling in the tumor cells with respect to osteolytic metastasis, we injected tumor cells via the left ventricle and assessed the incidence of osteolytic metastases at the tibio-femoral junction 5 weeks later. In the mice injected with 435-vector control cells, we saw metastasis in 100% of the mice injected. In contrast, relative to the mice injected with control cells, the incidence in mice injected with tumor cells stably silenced for GLI1 was reduced to 60% (each knee is scored as an incident; **Figure 7** **and Figure S2C**). Overall, there was a decrease in the intensity of the osteolytic metastasis as well. The data suggests that Hh signaling in the tumor cells is essential to the development of osteolytic metastases. These cells are also capable of directly activating osteoclast differentiation (assessed by TRAP staining) and stimulating resorption activity (**Figures S2D & E**). Moreover, the active Hh signaling and expression of OPN are important attributes for the tumor cells to activate osteoclast differentiation and resorptive activity. Thus, the data suggests that Hh signaling in the tumor cells can directly impact the ability of the cells to cause osteolysis.

## Discussion

The Hh pathway plays an essential function in regulating cell fate and in developmental patterning in animals and humans. This pathway is also important in the formation of the skeleton. During skeletogenesis and endochondral ossification Hh signaling coordinates growth and differentiation [21], [22], [23]. In adult animals, systemic administration of the ligand SHH, resulted in a primary increase in osteoblasts and their precursors. Interestingly, this was accompanied by an enhanced osteoclastogenic potential and decreased bone volume due to upregulation of the PTH/PTHrP receptor. Thus, Hh signaling in the adult bone milieu caused stimulatory effects on osteoprogenitors and osteoblasts resulting in bone remodeling and reduced bone strength because of a secondary increase in osteoclastogenesis [24].

The bone is a common site of metastasis for several malignancies. The impact of metastasized tumor cells in the bone disrupts the balance between the activities of the osteoclasts and osteoblasts. Radiographically, the bone lesions are classified as being osteolytic (bone loss) or osteosclerotic (bone formation) or mixed. Breast cancer bone metastases are usually osteolytic, characterized by excess bone turnover and consequent bone resorption. This is concomitant with the apoptosis and elimination of osteoblasts. In fact, several papers suggest that breast cancer cells limit osteoblasts by either inducing apoptosis or interfering with normal function and thus facilitating osteolysis through increased osteoclast activity [11], [25], [26], [27]. Paradoxically, it must be noted that the basic trigger for the differentiation of pre-osteoclasts to osteoclasts is supplied by the osteoblasts. Osteoblasts produce M-CSF and RANKL that promote pre-osteoclasts to differentiate into multinuclear, activated osteoclasts that adhere to bone and degrade the bone matrix [18]. RANKL and M-CSF activate a dendritic cell-specific transmembrane protein (DC-STAMP) that facilitates cell-cell adhesion and cytoskeletal re-arrangements resulting in a multinucleate osteoclast [28]. Thus, the availability of differentiated osteoblasts is vital to the development of active osteoclasts. Likewise, osteoclasts express BMPs that promote recruitment and proliferation of osteoblasts at resorption sites [19].

Thus, given the vital role that osteoblasts play in facilitating osteoclast activity, the elimination of osteoblasts by the tumor cells seems counter intuitive and warrants further understanding of the delicate balance between osteoblast and osteoblasts. In this study, we have investigated the role of Hh signaling in tumor cells on the interaction between tumor cells and osteoblasts. We have determined that breast cancer cells express elevated staining intensities for the Hh ligand IHH and the transcription factor, GLI1, indicating that the Hh pathway is activated in breast tumor cells. The upregulated expression of Hh ligands and GLI1 has also been studied by other investigators. In studies from independent laboratories, the expression of GLI1 and Hh ligand(s) has been shown to be indicative of activated Hh signaling and is associated with poorly differentiated phenotype and increased metastatic ability [29]. This has been found to be reported in multiple malignancies. As recently reported by O'Toole and others [30], a paracrine signature, encompassing high epithelial Hh ligand and high stromal GLI1, was an independent predictor for overall survival in multivariate analysis. Specifically, expression of IHH, PTCH and GLI1/2 has been determined to be correlated with enhanced proliferation in invasive breast carcinoma, node metastasis and clinical stage of breast cancer [31]. In other studies, the nuclear localization of GLI1 has been found to be associated with the expression of estrogen receptor-α [32], [33]. Upregulated GLI1 expression was also found to be a reliable indicator of constitutive Hh activation in surgically-resected ER-α-negative breast cancer patients [34]. Thus, our observations are in agreement with previous reports and indicate a constitutive Hh signaling in advanced breast cancer.

In order to determine the consequences of the interaction between tumor cells and osteoblasts, we investigated osteoblast differentiation at early (14 days) and late (21 days) post-initiation of differentiation in presence of conditioned media from tumor cells. While Hh ligands expressed by the tumor cells enhanced osteoblastogenesis and mineralization activity as an early event, we also observed enhanced expression of osteoclastogenesis-promoting factors viz. RANKL and PTHrP in the differentiated osteoblasts. Likewise, OPN expressed by the tumor cells also stimulated osteoblast differentiation. Tumor cells with a competent Hh pathway were more potent at inducing osteoblast differentiation and expression of RANKL and PTHrP.

While the expression of osteoblast differentiation markers, BSP and osteocalcin dwindled at a later event (21 days) characterized by increased apoptosis of the osteoblasts, the expression of RANKL and PTHrP continued to be robust, suggesting that the osteoblasts were expressing factors that would propel osteoclastogenesis. Thus, our data suggest that tumor cells initially enhance the differentiation of osteoblasts that in turn, express osteoclastogenesis enhancing factors. Later, as the osteoblasts get eliminated, the availability of RANKL and PTHrP creates an environment that will stimulate osteoclast differentiation and activity. Thus, an active Hh signaling in the tumor cells facilitates the generation of an osteoclast-stimulating milieu by initially kick-starting osteoblast development. This is apparent in the fact that ablating GLI1 severely compromised the ability of the tumor cells to form osteolytic metastasis in an experimental model of bone metastasis.

Miao *et al* [35], [36] have previously reported a role for osteoblast-derived PTHrP as a physiological regulator of bone remodeling. PTHrP is produced by cells of early osteoblast lineage that do not express PTH-receptor. PTHrP acts on receptor-positive committed preosteoblasts, and these cells respond by differentiating into mature osteoblasts. PTHrP acts directly on mature osteoblasts and osteocytes to prevent their apoptosis and is also required to enhance production of RANKL by PTHR1-positive pre-osteoblasts. As a result, osteoclast formation is promoted by interaction of the membrane molecule, RANKL, with its receptor, RANK. It is surmised that a fine balance or spatiotemporal control mechanisms exist to ensure availability of PTHrP for enhancing osteoblast differentiation, as persistently increased local PTHrP levels would favor increased osteoclast formation, through stimulation of RANKL production resulting in increased bone resorption, and high-turnover osteoporosis [19]. In fact, our results show a steady expression of PTHrP by osteoblasts (at 21 days) and are supported by the fact that Hh signaling competent tumor cells in fact, cause radiographically evident osteolysis in animal models.

Skeletal integrity is an essential survival function of mammals. Our findings reveal that the tumor cells can alter the balance between the activities of osteoblasts and osteoclasts via Hh signaling. Thus, given the fact that breast cancer cells express Hh ligands (**Figure S1**) and that Hh signaling propels breast cancer progression [33], [37], [38], [39], our studies imply that administration of pharmacological Hh inhibitors can inhibit Hh signaling in breast cancer cells, osteoblasts and osteoclasts and may reduce breast cancer-mediated bone loss in metastatic disease. This strategy targets the tumor cells as well as the bone and its microenvironment [40] and can reduce tumor burden and tumor-derived bone lesions.

## Supporting Information

Figure S1 (**A**) **To visualize differentiated osteoblasts, cells were stained with Alizarin Red and wells scanned at the end of 14 days of differentiation process.** Shown are representative well scan images and photomicrographs of differentiated osteoblasts. A representative mineralized nodule is encircled. **(B) The Hh pathway inhibitor, cyclopamine restricts GLI1 to the cytosol**. hFOB cells were cultured in absence (control) or in presence of cyclopamine (20 µM) for 24 h. The cells were fixed in 4% formaldehyde, permeabilized in 0.5% Triton-X and probed with anti-GLI1 antibody followed Alexa.Fluor 488-coupled second antibody (Molecular Probes). Cells were observed under either DIC or fluorescence (at 488 nm for Alexa.Fluor) and 461 nm for DAPI. Photomicrographs were acquired at using Axiovert 200 M Fluorescence Microscope (Zeiss). In the composite shown, GLI1 is stained green. **(C) Cyclopamine treatment causes GLI1 to accumulate in the cytosol**. Nuclear and cytosolic fractions were prepared after treating hFOB cells with cyclopamine. HDAC1 is used as a marker of purity of the nuclear fraction. **(D) Hh ligands produced by the tumor cells upregulates expression of BSP and osteocalcin in the osteoblasts 14 days after initiation of differentiation**. Deprivation of the Hh ligands from the tumor cell-conditioned medium using the 5E1 neutralizing antibody caused a significant reduction in the levels of BSP (SUM1315+5E1: ∧p = 0.01; 435+5E1: ∧p<0.0001) and osteocalcin (OC) (SUM1315+5E1: ∧p = 0.001; 435+5E1: ∧p<0.0001).
(TIF)

Figure S2 **(A) Expression of GLI1 and OPN in the tumor cells enhances their ability to induce RANKL and PTHrP by the osteoblasts**. Abrogation of GLI1 expression in the SUM1315 cells reduces the expression of RANKL (KD2:†p = 0.02) and PTHrP (KD2: †p = 0.02003) elicited by the conditioned media from these cells. Likewise, conditioned medium from MDA-MB-435 cells abrogated for GLI1 was less efficient at inducing expression of RANKL (KO1: †p<0.0001) and PTHrP (KO1: †p<0.0001) by the osteoblasts. Ablating expression of OPN also caused a significant reduction in eliciting the expression of RANKL (OPNi: †p<0.0001) and PTHrP (OPNi: †p<0.0001) in osteoblasts. **(B) Extended differentiation in presence of conditioned media from tumor cells promotes osteoblast apoptosis**. The extent of apoptosis was assessed by TUNEL staining followed by DAPI and phalloidin counterstaining. Representative images shown depict apoptosis recorded for **a**: DM; **b**: SUM1315; **c**: MDA-MB-231; **d**: SUM159; **e**: MDA-MB-435. **(C) Abrogating GLI1 expression reduces the incidence and intensity of osteolysis inflicted by MDA-MB-435 cells**. Radiographic images **(i)** and **(ii)** represent osteolysis in mice injected with MDA-MB-435-vector control cells. Images **(iii)** and **(iv)** represent absence of evidence of osteolysis in mice injected with MDA-MB-435-KO1 (silenced for GLI1) cells. Cells were injected via the intracardiac route. **(D) Interfering with Hh signaling decreases with the ability of tumor cells to induce osteoclast differentiation**. Relative to DM, the conditioned medium from the MDA-MB-435 cells causes the development of significantly increased numbers of TRAP-positive multinucleate osteoclasts (†p = 0.0004). There was a significant reduction in this ability following interference with Hh signaling in the tumor cells with cyclopamine treatment (∧p<0.0001) or silencing GLI1 (∧p<0.0001). Silencing OPN from the tumor cells also significantly reduced (∧p = 0.001) their ability to elicit osteoclast differentiation. Osteoclast differentiation was scored using the TRAP assay following the manufacturer's protocol (Sigma). **(E) Interfering with Hh signaling decreases with the ability of tumor cells to enhance resorptive activity of osteoclasts**. Conditioned medium from the MDA-MB-435 cells significantly enhances (†p = 0.006) the ability of DM to induce resorptive activity of osteoclasts. Cyclopamine treatment (∧p = 0.007) or GLI1-silencing (∧p = 0.016) or OPN-silencing (∧p = 0.03) of the tumor cells significantly reduced their ability to activate the resorptive function of osteoblasts. The ability of the osteoclasts to resorb bone matrix was tested using osteoclast activity assay (OAAS plates, Osteogenic Core Technologies).
(TIF)
