# Supplementary figures and images for: Hedgehog Signaling in Tumor Cells Facilitates Osteoblast-Enhanced Osteolytic Metastases
Source: PLoS One. 2012 Mar 29;7(3):e34374. doi: 10.1371/journal.pone.0034374 (PMC3315536; doi:10.1371/journal.pone.0034374)

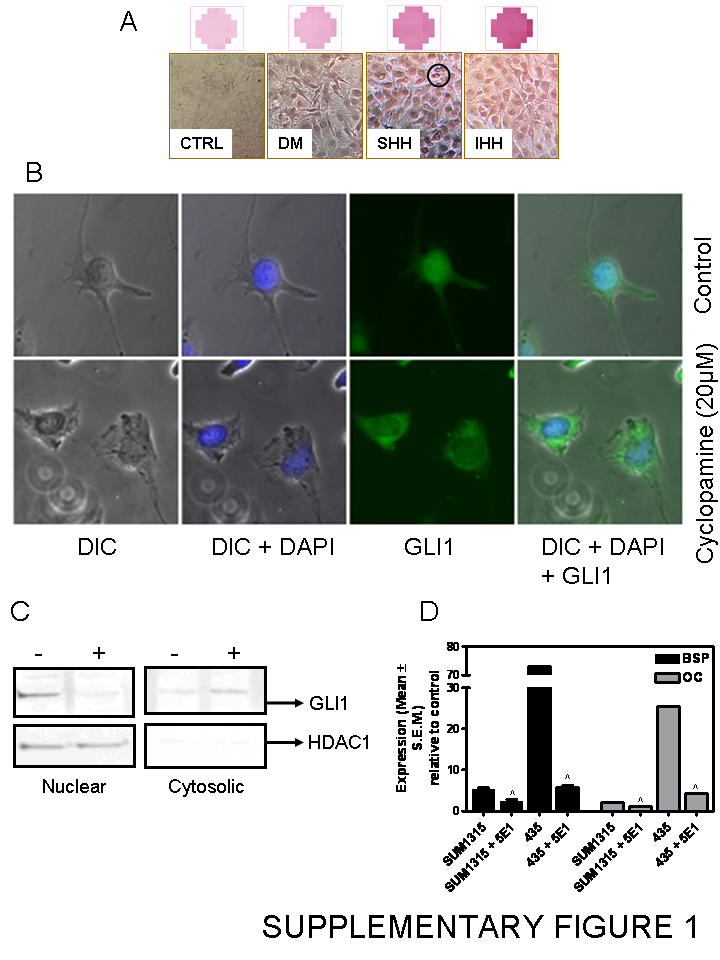

Supplement: Figure S1 — (A) To visualize differentiated osteoblasts, cells were stained with Alizarin Red and wells scanned at the end of 14 days of differentiation process. Shown are representative well scan images and photomicrographs of differentiated osteoblasts. A representative mineralized nodule is encircled. (B) The Hh pathway inhibitor, cyclopamine restricts GLI1 to the cytosol. hFOB cells were cultured in absence (control) or in presence of cyclopamine (20 µM) for 24 h. The cells were fixed in 4% formaldehyde, permeabilized in 0.5% Triton-X and probed with anti-GLI1 antibody followed Alexa.Fluor 488-coupled second antibody (Molecular Probes). Cells were observed under either DIC or fluorescence (at 488 nm for Alexa.Fluor) and 461 nm for DAPI. Photomicrographs were acquired at using Axiovert 200 M Fluorescence Microscope (Zeiss). In the composite shown, GLI1 is stained green. (C) Cyclopamine treatment causes GLI1 to accumulate in the cytosol. Nuclear and cytosolic fractions were prepared after treating hFOB cells with cyclopamine. HDAC1 is used as a marker of purity of the nuclear fraction. (D) Hh ligands produced by the tumor cells upregulates expression of BSP and osteocalcin in the osteoblasts 14 days after initiation of differentiation. Deprivation of the Hh ligands from the tumor cell-conditioned medium using the 5E1 neutralizing antibody caused a significant reduction in the levels of BSP (SUM1315+5E1: ∧p = 0.01; 435+5E1: ∧p<0.0001) and osteocalcin (OC) (SUM1315+5E1: ∧p = 0.001; 435+5E1: ∧p<0.0001). (TIF) [file pone.0034374.s001.tif]

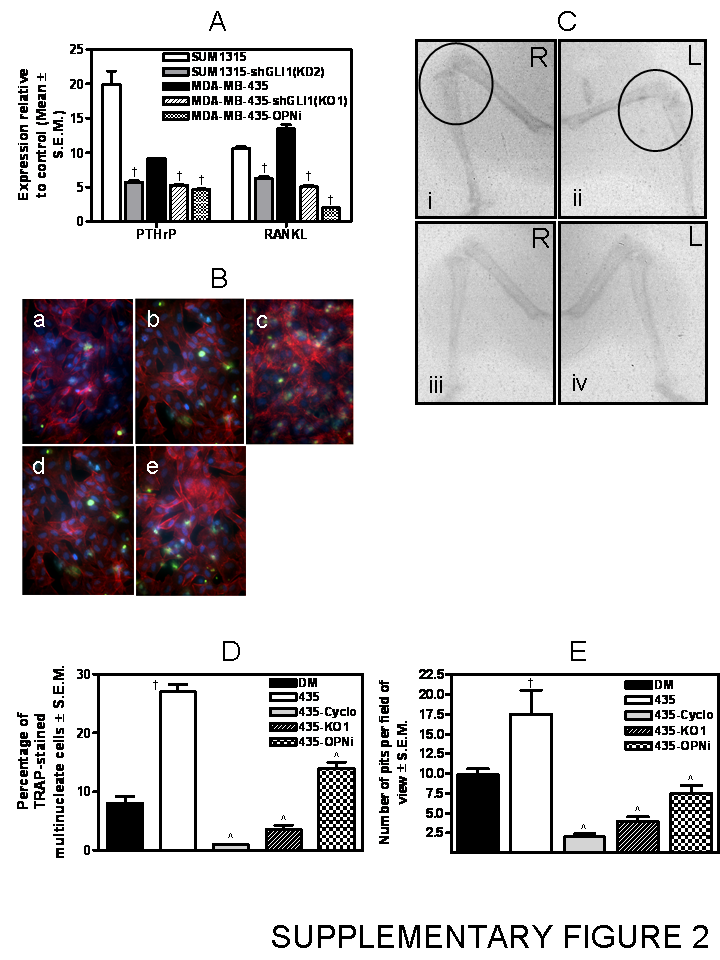

Supplement: Figure S2 — (A) Expression of GLI1 and OPN in the tumor cells enhances their ability to induce RANKL and PTHrP by the osteoblasts. Abrogation of GLI1 expression in the SUM1315 cells reduces the expression of RANKL (KD2:†p = 0.02) and PTHrP (KD2: †p = 0.02003) elicited by the conditioned media from these cells. Likewise, conditioned medium from MDA-MB-435 cells abrogated for GLI1 was less efficient at inducing expression of RANKL (KO1: †p<0.0001) and PTHrP (KO1: †p<0.0001) by the osteoblasts. Ablating expression of OPN also caused a significant reduction in eliciting the expression of RANKL (OPNi: †p<0.0001) and PTHrP (OPNi: †p<0.0001) in osteoblasts. (B) Extended differentiation in presence of conditioned media from tumor cells promotes osteoblast apoptosis. The extent of apoptosis was assessed by TUNEL staining followed by DAPI and phalloidin counterstaining. Representative images shown depict apoptosis recorded for a: DM; b: SUM1315; c: MDA-MB-231; d: SUM159; e: MDA-MB-435. (C) Abrogating GLI1 expression reduces the incidence and intensity of osteolysis inflicted by MDA-MB-435 cells. Radiographic images (i) and (ii) represent osteolysis in mice injected with MDA-MB-435-vector control cells. Images (iii) and (iv) represent absence of evidence of osteolysis in mice injected with MDA-MB-435-KO1 (silenced for GLI1) cells. Cells were injected via the intracardiac route. (D) Interfering with Hh signaling decreases with the ability of tumor cells to induce osteoclast differentiation. Relative to DM, the conditioned medium from the MDA-MB-435 cells causes the development of significantly increased numbers of TRAP-positive multinucleate osteoclasts (†p = 0.0004). There was a significant reduction in this ability following interference with Hh signaling in the tumor cells with cyclopamine treatment (∧p<0.0001) or silencing GLI1 (∧p<0.0001). Silencing OPN from the tumor cells also significantly reduced (∧p = 0.001) their ability to elicit osteoclast differentiation. Osteoclast different [file pone.0034374.s002.tif]
